# Temporal Shifts in Climate-Influenza Dynamics: A Multi-Subtype Analysis in Southern China Spanning the COVID-19 Era

**DOI:** 10.1155/tbed/5581162

**Published:** 2025-08-11

**Authors:** Lirong Zhang, Jiajia Lin, Jiaxiong Zheng, Yixiao Niu, Fancun Meng, Suyang Liu, Qiaocheng Chang, Zicheng Cao

**Affiliations:** ^1^School of Public Health, Shantou University, Shantou 515041, China; ^2^State Key Laboratory of Public Big Data, Guizhou University, Guiyang 550025, China; ^3^School of Public Health (Shenzhen), Sun Yat-sen University, Shenzhen Campus of Sun Yat-sen University, Shenzhen 518107, China; ^4^Shenzhen Key Laboratory of Pathogenic Microbes and Biosafety, Sun Yat-sen University, Shenzhen Campus of Sun Yat-sen University, Shenzhen 518107, China

**Keywords:** climate-influenza interactions, COVID-19 pandemic, Southern China, temporal dynamics

## Abstract

**Background:** The COVID-19 pandemic disrupted global influenza transmission. We aimed to elucidate how meteorological and air pollution drivers influenced seasonal influenza A subtypes and B lineage in Southern China pre-, during-, and postpandemic.

**Methods:** We analyzed weekly influenza surveillance data from Southern China (2011–2024) and corresponding meteorological data. Using an interpretable machine learning framework combining XG-Boost and SHapley Additive exPlanation (SHAP), we quantified the dynamic contributions of environmental factors to influenza subtype positivity rates. GAM models were employed to identify climate thresholds for influenza transmission.

**Findings:** The pandemic reshaped influenza transmission and environmental dependencies in Southern China. Post-pandemic circulation showed marked subtype‑specific shifts: A/H3N2 winter positivity nearly tripled (12.670% vs. 4.861% pre-pandemic, *p*  < 0.001), A/H1N1 increased over four‑fold (4.325% vs. 0.935% pre-pandemic, *p* = 0.064), while B/Victoria circulation declined significantly (0.594% vs. 1.623% pre-pandemic, *p*  < 0.001). Environmental driver hierarchies underwent notable temporal reorganization. For A/H3N2, PM_10_ influence surged from 9.18% to 30.37% during the pandemic before dropping to 3.95% post-pandemic, while visibility emerged as the dominant driver (58.05% vs. 37.26% pre-pandemic). A/H1N1 showed peak humidity sensitivity during the pandemic (18.59%) that later diminished (3.78%), with transmission promotion occurring only below a threshold of ≤11.44 g/m³ absolute humidity. B/Victoria maintained consistent sea‑level pressure sensitivity, peaking post-pandemic (36.36%), with transmission optima at 8.58 g/m³. Seasonal modeling revealed subtype and phase‑specific environmental thresholds that shifted between “promotion” and “inhibition” zones for humidity and visibility, indicating key alterations in climate‑influenza relationships across the pandemic transition.

**Interpretation:** These findings show that pandemic‑era disruptions recalibrated the environmental architecture of influenza transmission. New environmental thresholds and driver hierarchies reveal shifts in viral‑climate relationships. The identification of distinct “promotion‑inhibition” zones for each subtype establishes a new paradigm for climate‑pathogen interactions. These insights require immediate integration into surveillance frameworks and predictive models.

## 1. Introduction

Seasonal influenza poses a significant public health burden in China, contributing substantially to morbidity, mortality, and economic losses. The annual impact of influenza epidemics in China is estimated to result in 88,100 respiratory and circulatory excess deaths, with an associated economic burden of approximately US$6.0 billion. In the wake of the COVID-19 pandemic, Southern China has experienced notable shifts in seasonal influenza transmission patterns [1]. Recent studies have observed an overall higher positivity rate for influenza viruses in the post-pandemic period compared to pre-pandemic levels, suggesting potential alterations in viral ecology and transmission dynamics [2]. Moreover, the differential rebound of specific subtypes—particularly the resurgence of A/H3N2 and A/H1N1 versus the continued low activity of B/Yamagata—highlights the need for subtype‑focused analyses. This phenomenon warrants closer examination to understand the factors driving these epidemiological changes and their implications for public health strategies.

The role of environmental factors in influencing seasonal influenza transmission has been well-documented, with temperature, humidity, and other meteorological variables playing crucial roles in viral survival, transmission efficiency, and host susceptibility [3–5].

While temperature and humidity relationships are well-established in temperate regions, tropical and subtropical environments present distinct challenges due to their diverse seasonal patterns and complex climatic dynamics [6]. Recent evidence suggests that air quality metrics—including PM_2_._5_ and visibility—significantly modulate influenza risk in these settings, yet remain poorly integrated with traditional meteorological drivers in predictive frameworks. Seasonal influenza epidemics are driven by genetically and antigenically distinct viral populations: Two influenza A subtypes (A/H3N2 and A/H1N1) and two influenza B lineages (B/Victoria and B/Yamagata). Notably, B/Yamagata has virtually disappeared globally since March 2020, leaving B/Victoria as the predominant influenza B lineage [7]. These viral types exhibit markedly different environmental sensitivities—while both influenza A and B typically peak during winter and spring, A/H3N2 may also show pronounced summer circulation in subtropical regions [8]. However, most epidemiological studies have analyzed combined influenza data or focused on influenza A viruses, which dominate most seasons, creating significant knowledge gaps regarding type-specific environmental relationships.

Southern China's mainland features diverse terrains and a subtropical monsoon climate, characterized by high annual precipitation, especially in summer, and frequent typhoons contributing to a humid environment. The region's warm, humid winters and dense population facilitate active influenza virus spread year-round [9]. This unique environmental context, coupled with the region's demographic characteristics, makes Southern China an ideal setting for studying the nuanced relationships between climate factors and influenza subtype transmission. The COVID‑19 pandemic introduced unprecedented disruptions via stringent nonpharmaceutical interventions (NPIs), including mobility restrictions, mask mandates, and indoor ventilation changes, which in turn altered both environmental exposures and host contact patterns [10]. These interventions have potentially reshaped the relationship between environmental factors and influenza infection risk, necessitating a reevaluation of our understanding of these dynamics in the postpandemic era.

This study aims to elucidate the complex interplay between environmental factors and the transmission dynamics of influenza subtypes A/H3N2, A/H1N1, and B/Victoria lineage in Southern China across three distinct phases: prepandemic, during the pandemic, and postpandemic. By stratifying our analysis across these periods, we seek to capture the temporal shifts in the influence of climatic and air‑quality variables on influenza transmission, particularly in light of the significant perturbations caused by NPIs. Our approach employs advanced machine learning techniques, including XG-Boost models and the SHapley Additive exPlanation (SHAP) framework [11], to quantify the contributions of various environmental drivers—temperature, absolute humidity, sea‑level pressure, visibility, and PM_10_—to subtype‑specific influenza positivity rates. Furthermore, we apply generalized additive models (GAMs) to detect nonlinear and threshold‑dependent effects, thereby identifying critical environmental cutoffs that may trigger changes in viral transmission risk. This methodology allows us to disentangle the separate and interactive roles of climate change and pandemic interventions on influenza subtype activity, addressing a critical gap in our understanding.

Our findings contribute to a nuanced understanding of how environmental factors modulate influenza transmission in the context of major public health disruptions. By revealing subtype-specific sensitivities to environmental variables and their temporal shifts, this research provides valuable insights for tailoring surveillance strategies, forecasting models, and intervention measures in the postpandemic era. Ultimately, this study aims to enhance our ability to anticipate and mitigate future influenza outbreaks in the face of evolving environmental and public health landscapes, particularly in regions with complex seasonal influenza patterns like Southern China.

## 2. Materials and Methods

### 2.1. Influenza Data

Influenza surveillance data were obtained from the China National Influenza Center (CNIC) (https://ivdc.chinacdc.cn/cnic/) and covered the period from January 1, 2011 to May 1, 2024. This dataset included weekly counts of influenza virus detections and positive cases for the major circulating subtypes: A/H3N2, A/H1N1, and B/Victoria, across Southern China, defined by cities located south of the Qinling-Huaihe line. The positivity rate for each influenza subtype was calculated using the following formula:

  $$\begin{array}{c} P_{i,t}=\frac{N_{i,\text{⁣}t}}{N_{\text{total},\text{⁣}t}}\times 100\%, \end{array}$$

where *P*_*i*,*t*_ represents the positivity rate of influenza subtype *i* (e.g., A/H3N2, A/H1N1, and B/Victoria) at time *t*, *N*_*i*,⁣*t*_ is the number of positive cases for subtype *i* at time *t* and *N*_total,⁣*t*_ is the total number of samples tested at time *t*.

To analyze the effect of environmental factors on influenza subtype transmission, we divided the study period into three distinct phases, based on the timeline of the COVID-19 pandemic in China:

1. Prepandemic period (January 5, 2011–December 25, 2019): Defined as the time before the detection of SARS-CoV-2 in China, during which no COVID-19 cases or nationwide public health interventions occurred.
2. Pandemic period (January 1, 2020–October 26, 2022): Corresponding to the period of confirmed COVID-19 transmission and implementation of the dynamic zero-COVID policy (including border controls, lockdowns, and mass testing). The start date aligns with the first nationwide case reports and stringent public health responses; the end date represents the last complete week before any optimization of those measures.
3. Postpandemic period (November 2, 2022–May 1, 2024): Defined by the initial relaxation of China's dynamic zero-COVID policy, supported by two lines of evidence. First, in early November 2022 the State Council called out “one-size-fits-all” and overly stringent controls, and on November 11 the National Health Commission rolled out a 20-point optimization plan—measures, such as home isolation for mild cases, reduced testing requirements, and precision rather than blanket lockdowns—that were rapidly adopted in southern cities, alongside phased removal of roadblocks and decentralization of community controls. Second, because our analysis uses weekly case data and policy shifts unfolded over several days, November 2, 2022 (the first day of the first full postrelaxation week) was selected as the transition point, thereby capturing the collective weekly response and avoiding the arbitrariness of a single-day breakpoint.

### 2.2. Meteorological Data

Meteorological data were obtained from 111 prefecture-level cities in Southern China via the visual crossing weather service. The data covered daily values for 13 climatic factors, including temperature (°C), diurnal temperature range (DTR, °C), relative humidity (%), visibility (km), sea-level pressure (hPa), solar radiation (W/m^2^), cloud cover (%), absolute humidity (g/m^3^), wind speed (m/s), wind direction (degrees), solar energy (kWh/m^2^), UV index, and “feels like” temperature (°C) (See Supporting Information 1–Supporting Information 3 for raw values). Absolute humidity (AH, %) was computed using the Clausius-Clapeyron equation [12] (see Supporting Information 4 for details). Additionally, atmospheric pressure values were adjusted to sea level to standardize comparisons across cities with varying altitudes.

While visibility was initially included as a proxy for atmospheric conditions, it is influenced by multiple factors (e.g., humidity, fog, and aerosols) and does not specifically capture pollutant exposure [13]. To address this, we incorporated key air pollutants as additional variables. Given evidence linking air pollution to respiratory viral infections, such as influenza [14], we included daily concentrations of PM_2_._5_, PM_10_, and NO_2_ from the National Tibetan Plateau Data Center, aligned with meteorological data in both spatial and temporal resolution (Supporting Information 4: Table S6). All values were measured in μg/m^3^.

### 2.3. Data Preprocessing

Both the influenza and meteorological data were aggregated into weekly time series for subsequent analysis. The influenza data were recorded as weekly counts, while the daily meteorological data were averaged over 7-day intervals to generate weekly means for each meteorological factor. A matching table was established to align the weekly meteorological data with the positivity rates of influenza A subtypes and B lineage, calculated based on the total number of positive cases detected in Southern China. Prior to modeling, all time series were checked for missing data, and any missing values were imputed using linear interpolation where necessary, ensuring consistency across datasets for accurate analysis.

### 2.4. Machine-Learning Framework

To model the relationship between temporal environmental variables and the positivity rates of various influenza A subtypes and B lineage, we constructed individual extreme gradient boosting (XG-Boost) models for each subtype. XG-Boost was chosen for its superior handling of structured data and time series [15], particularly its ability to manage missing data and capture complex, nonlinear interactions between predictors. Each climatic variable, including temperature, relative humidity, visibility, and other factors, were treated as an explanatory feature. The target variable for each model was the positivity rate of the corresponding influenza subtype.

Model performance was evaluated using several metrics, including the coefficient of determination (*R*^2^), root-mean-square error (*RMSE*), mean square error (*MSE*), and mean absolute error (*MAE*), ensuring robust validation of each model's predictive power (see Supporting Information 4: Table S1 for detailed results).

To interpret the contributions of individual climatic variables, we employed SHAP [16], a method that quantifies the influence of each feature on model predictions. SHAP values were computed for each observation, reflecting the positive or negative impact of each meteorological factor on influenza subtype positivity rates. These values decompose the total prediction into contributions from all features, allowing for a granular, instance-level interpretation of the relationships between environmental factors and influenza transmission dynamics. The SHAP values were calculated as follows:

  $$\begin{array}{c} f\left(x\right)=\varphi_{0}+\sum_{i=1}^{M}\varphi_{i}x_{i}, \end{array}$$

where *f*(*x*) is the prediction for a specific instance, *φ*_0_ is the baseline prediction (average across all samples), and *φ*_*i*_ is the contribution of feature *x*_*i*_ to the final prediction, with *M* representing the total number of features.

We further stratified SHAP analyses by prepandemic and postpandemic periods. The SHAP values were averaged for each time period and visualized through summary plots, enabling the identification of temporal shifts in the influence of key environmental factors on subtype positivity rates (See Supporting Information 1–Supporting Information 3 for feature SHAP values for influenza A subtypes and the B lineage).

Additionally, to assess the nonlinear effects of environmental variables on the infection risk of each influenza subtype, we modeled the relationship between the SHAP values (representing the marginal contribution of each environmental variable to the predicted risk) and the corresponding variable values using GAMs implemented via the pygam package [17]. Smoothing parameters (number of splines and penalty) were optimized for each influenza subtype and time period based on prior tuning (Supporting Information 4: Table S2). We identified thresholds for key environmental variables as the points where the GAM partial dependance curves of SHAP values crossed zero, indicating a shift in the direction of risk contribution. To quantify the uncertainty of these thresholds, we performed bootstrap resampling (150 iterations), refitting the GAM, and recalculating crossing points in each bootstrap sample to derive 95% confidence intervals. Together, the XG-Boost and SHAP framework, augmented by GAM analysis with bootstrap validation, provided a robust and interpretable assessment of how environmental factors influenced the transmission dynamics of influenza A subtypes and B lineage pre-, during-, and post-pandemic.

### 2.5. Statistical Analysis

All statistical analyses were conducted using Python (version 3.9.0). Continuous variables were summarized as medians with interquartile ranges (IQRs). Group comparisons were performed using either the independent *t*-test for normally distributed data or the Mann–Whitney *U* test for nonparametric data, as appropriate. Statistical significance was defined as *p*  < 0.05, with all tests conducted under a 95% confidence level.

## 3. Results

### 3.1. XGBoost Model Performance Evaluation

Supporting Information 4: Table S1 shows that our XGBoost models achieved robust explanatory performance across B/Victoria, A/H1N1, and A/H3N2. *R*^2^ values exceeded 0.90 for all three lineages, *MSE* ranged from 0.0030 to 0.0166, *RMSE* from 0.0544 to 0.1288 % points, and *MAE* from 0.0365 to 0.0916% points. The A/H3N2 model exhibited slightly higher errors, likely due to its stronger seasonal variability, whereas the B/Victoria and A/H1N1 models showed lower error metrics. Overall, *RMSE* and *MAE* remained below 0.10% points, indicating that fitting errors fall within the surveillance noise floor.

### 3.2. Epidemiological Dynamics of Seasonal Influenza in Southern China

Before the COVID-19 pandemic, influenza circulation in Southern China was typically dominated by one subtype, although several subtypes co-circulated. Alternating predominance was observed, with A/H1N1 prevailing in 2013–2014 and 2018–2019, and A/H3N2 dominating in 2014–2015 and 2017–2018. Notably, A/H3N2 demonstrated a biannual peak circulation pattern, with year-round activity, while A/H1N1 and B/Victoria predominantly circulated in the summer. However, during the COVID-19 pandemic (2021–2022), only B/Victoria was observed, exhibiting year-round circulation. In the post-pandemic phase, single-subtype dominance returned with A/H3N2 being the sole circulating strain in 2022–2023, and in the summer of 2023, only influenza A viruses were detected, with no influenza B circulation. As national control measures were lifted and pandemic management transitioned to a normalized phase (2023–2024), multiple subtypes cocirculated again, with a notably higher circulation intensity compared to the pre-pandemic period.

**Figure 1:**
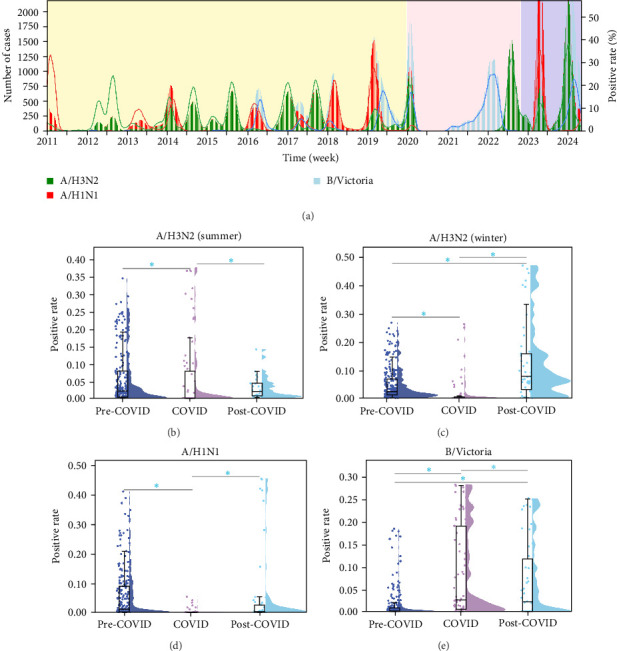
(A) Temporal trends in the number of cases and positive rates for the three influenza A subtypes and B lineage (A/H3N2, A/H1N1, B/Victoria) from 2011 to May 2024. (B–E) Distribution of positive rates across distinct periods: the pre-COVID era, COVID-19 era, and post-COVID era. *⁣*^*∗*^Indicates statistical significance (*p* < 0.05).

To further explore these epidemiological shifts, we analyzed subtype-specific positivity rates across three distinct phases: pre-pandemic, pandemic, and post-pandemic. Focusing on A/H3N2, which exhibited a year-round presence with dual peaks in summer and winter, we performed a seasonal comparison of positivity rates (Figure 1). Significant differences were observed in A/H3N2 positivity across the three periods (Figure 1B,C). In winter, the positivity rate for A/H3N2 was significantly higher post-pandemic (12.670%) compared to both the pre-pandemic (4.861%) and pandemic (1.829%) periods (*p*  < 0.001, Supporting Information 5). In summer, however, A/H3N2 positivity rates remained stable between the pre-pandemic (5.829%) and pandemic (5.808%) periods but declined post-pandemic (3.139%). Both A/H1N1 and B/Victoria displayed notable differences across the pandemic phases (Figure 1D/1E). A/H1N1 positivity rates showed a sharp increase post-pandemic (4.325%) compared to both the pre-pandemic (0.935%) and pandemic (0.005%) periods. However, no significant difference was detected between the pre- and post-pandemic periods (pre vs. post: *p* = 0.064, Supporting Information 4: Table S3). In contrast, B/Victoria's circulation exhibited a slight increase during the pandemic (1.750%) compared to pre-pandemic levels (1.623%), but positivity rates significantly declined post-pandemic (0.594%), indicating a contraction of B/Victoria's activity despite its extended circulation during the pandemic.

### 3.3. Environmental Factor Contributions to Subtype Incidence: Pre-, During, and Post-COVID-19

We observed substantial variations in environmental factor contributions across pandemic phases, with several factors showing pronounced changes that may have influenced transmission dynamics of different influenza subtypes and lineages. To systematically quantify these temporal shifts, we employed an XG-Boost model coupled with SHAP analysis to assess the relative contributions of environmental-climatic factors across prepandemic, pandemic, and postpandemic periods. Comprehensive factor contribution comparisons across all pandemic stages are detailed in Supporting Information 4: Table S5. We also performed comparative analyses of the raw values of each environmental factor across these stages, not solely relying on SHAP values (Supporting Information 4: Table S4).

**Figure 2:**
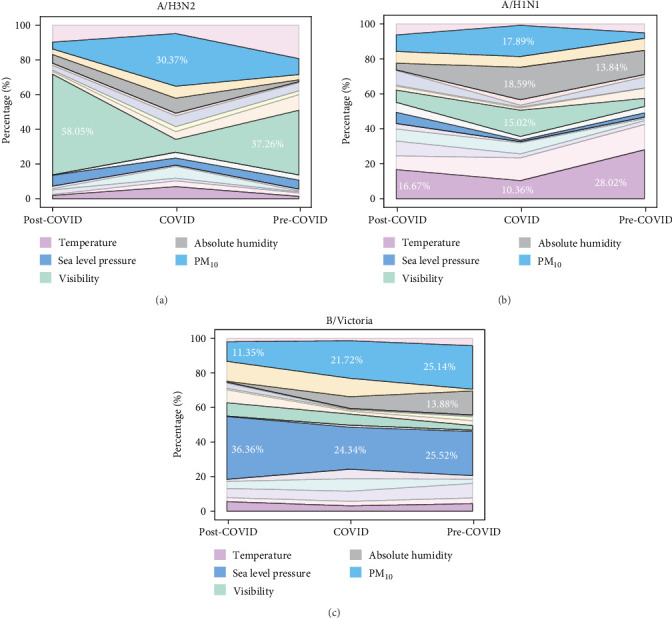
Contribution of environmental factors to A/H3N2 (A), A/H1N1 (B), and B/Victoria (C) dynamics across the pre-COVID, COVID-19, and post-COVID eras.

Analysis of A/H3N2 transmission dynamics revealed dramatic shifts in environmental factor importance (Figure 2A). PM_10_ emerged as a critical pandemic-period driver, with its relative contribution (measured by SHAP values) increasing more than three-fold from 9.18% pre-pandemic to 30.37% during the pandemic, before declining substantially to 3.95% post-pandemic. This pattern contrasted sharply with visibility, which exhibited an inverse relationship: its contribution decreased from 37.26% pre-pandemic to 7.42% during the pandemic, then surged to 58.05% post-pandemic—exceeding even pre-pandemic levels and becoming the dominant factor in the recovery phase. For A/H1N1 (Figure 2B), PM_10_ demonstrated a similar but less pronounced pattern, rising to 17.89% during the pandemic before moderating to 9.44% post-pandemic—nonetheless remaining elevated compared to the pre-pandemic baseline of 3.18%. Temperature showed a notable decline from 28.02% pre-pandemic to 10.36% during the pandemic, followed by partial recovery to 16.67% thereafter. Visibility exhibited increased importance during the pandemic (15.02%) before declining post-pandemic (7.02%), yet remained above initial levels. Absolute humidity peaked during the pandemic at 18.59% (from 13.84% pre-pandemic) before dropping markedly to 3.78% in the post-pandemic period.

B/Victoria lineage displayed distinctly different environmental dependencies (Figure 2C), with sea-level pressure maintaining consistent dominance throughout all phases. Its contribution remained relatively stable at 25.52% pre-pandemic and 24.34% during the pandemic, before increasing substantially to 36.36% post-pandemic. In contrast to the influenza A subtypes, both PM_10_ and absolute humidity showed progressive decline across all phases, decreasing from pre-pandemic values of 25.14% and 13.88% to post-pandemic minima of 11.35% and 0.49%, respectively.

### 3.4. Seasonal Patterns of Key Environmental Factor Influences on Subtype Incidence

**Figure 3:**
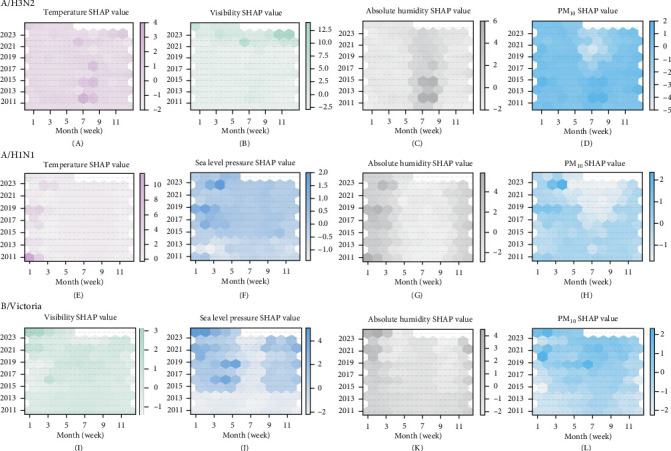
Temporal dynamics of key environmental factors (temperature, visibility, absolute humidity, sea-level pressure, and PM_10_) on the positive rates of A/H3N2, A/H1N1, and B/Victoria during different years (January 2011–May 2024). (A–L) illustrate the effects of the major environmental factors on the positive rates of the three influenza subtypes/lineages.

To identify the most influential variables, we selected five environmental drivers—visibility, PM_10_, temperature, absolute humidity, and sea-level pressure—because the sum of their SHAP contributions across B/Victoria, A/H1N1, and A/H3N2 ranked within the top five of all features (Supporting Information 4: Figure S1). We then examined the monthly mean SHAP values of these primary environmental drivers to characterize their evolving impacts on A/H3N2, A/H1N1, and B/Victoria infection risk across the three pandemic phases (Figure 3).

Temperature effects on A/H3N2 demonstrated marked seasonal and temporal variability. During the pre-pandemic period, temperature exhibited strong transmission-promoting effects in summer months (June–August, Figure 3A), consistent with established seasonal patterns. However, this promotional effect was substantially attenuated during the pandemic phase and showed only partial recovery in the post-pandemic period, remaining below pre-pandemic baseline levels. Visibility underwent a complete reversal in its influence profile: transitioning from mild transmission-inhibitory effects pre-pandemic to peak transmission-promoting effects during post-pandemic autumn months (October–December). PM_10_ generally maintained transmission-promoting effects throughout most periods, with a notable exception during pandemic summer months (June–September) when it transiently exhibited inhibitory effects (Figure 3D). Absolute humidity showed a progressive decline in its positive transmission effects over time across all pandemic phases. A/H1N1 transmission patterns revealed distinct environmental sensitivities. Temperature and humidity demonstrated strong transmission-promoting effects during early winter months (January–March) in the pre-pandemic period, but these effects progressively attenuated to near-neutral levels by the mid-pandemic phase (Figure 3E/3G). PM_10_ exhibited complex seasonal switching patterns: transitioning from mild inhibitory to promotional effects during January–March, while simultaneously shifting from mild promotional to inhibitory effects during May–September (Figure 3H). Sea-level pressure showed a notable transition from neutral or slightly inhibitory effects to clearly promotional effects during January–April across pandemic phases (Figure 3F).

B/Victoria lineage maintained its characteristic pressure-driven transmission profile throughout the study period. Sea-level pressure demonstrated a complex temporal pattern, shifting from inhibitory to promotional effects during January–April, before reverting to inhibitory effects during June–September in the post-pandemic period (Figure 3J). Visibility evolved from neutral or slightly inhibitory effects (January–March) to clearly promotional effects in the post-pandemic phase (Figure 3I). Absolute humidity showed increasingly promotional effects on infection during November–March periods, coupled with mild inhibitory effects during May–October (Figure 3K). PM_10_ exhibited a consistent evolution from January–March inhibitory effects to promotional effects across all subsequent periods and phases (Figure 3L).

### 3.5. Effect Relationships of Key Environmental Factors on Influenza Risk Transition

**Figure 4:**
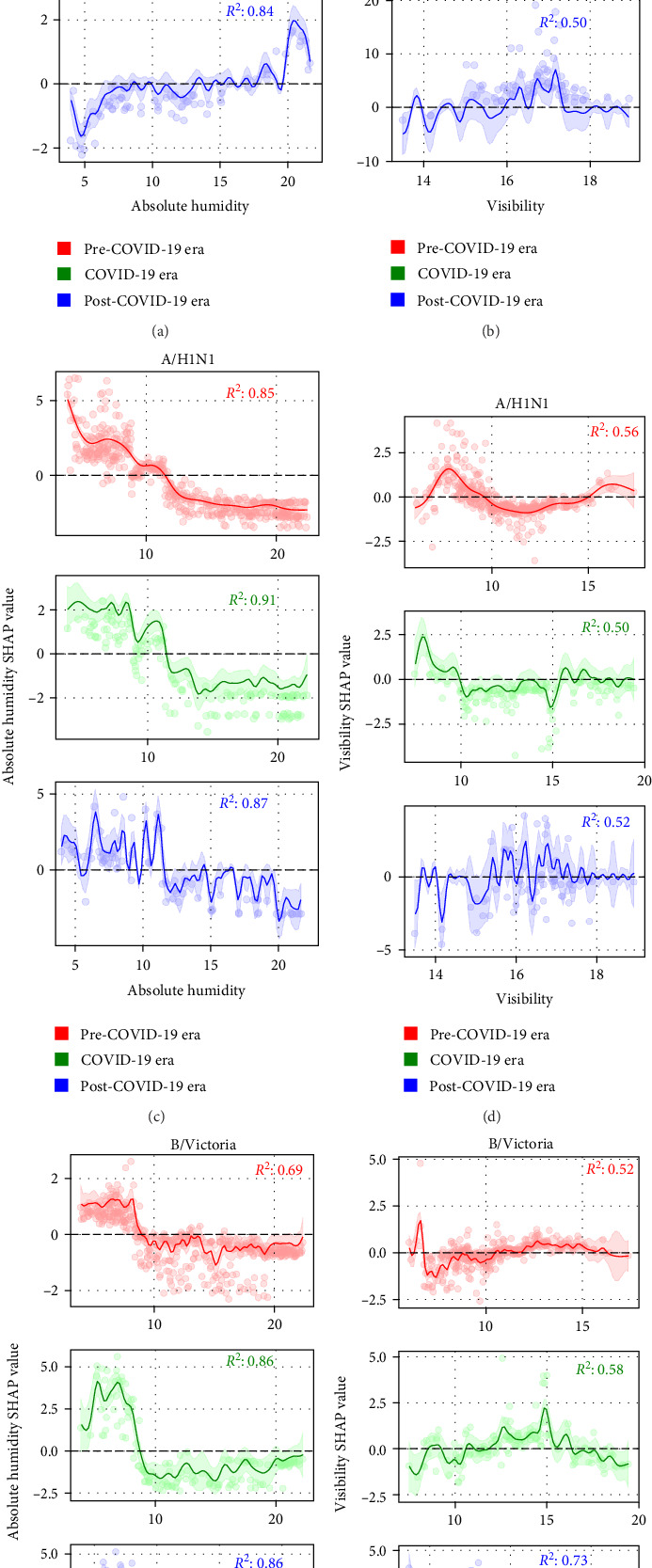
Nonlinear relationships between key environmental factors (visibility and absolute humidity) and the infection risks of A/H3N2, A/H1N1, and B/Victoria across the pre-COVID, COVID-19, and post-COVID eras. (A–F) illustrate the nonlinear effects of absolute humidity and visibility on the infection risks of the three influenza subtypes/lineages.

To quantify the threshold-dependent relationships between environmental drivers and subtype-specific infection risk, we employed GAMs to characterize how SHAP effects varied with key climatic variables across pandemic phases. This approach enabled identification of critical threshold transition points that governed risk switching for each influenza subtype (Figure 4).

Absolute humidity demonstrated distinct threshold-response patterns across influenza subtypes. For A/H3N2, all three pandemic phases exhibited a consistent biphasic relationship characterized by transmission promotion at high absolute humidity levels and inhibition at low levels. However, the critical threshold values showed temporal drift: 17.08 g/m³ (95% CI: 6.20–21.83 g/m³) pre-pandemic, declining to 14.93 g/m³ (95% CI: 14.48–15.58 g/m³) during the pandemic, then rising to 19.60 g/m³ (95% CI: 5.21–20.13 g/m³) post-pandemic. In marked contrast, both A/H1N1 and B/Victoria exhibited inverse threshold responses, with monotonic transmission promotion occurring only below specific humidity thresholds ≤11.44 g/m³ for A/H1N1 and ≤8.58 g/m³ for B/Victoria—and consistent inhibition above these critical values across all pandemic phases (Supporting Information 6).

Visibility effects revealed complex, phase-dependent threshold dynamics with substantial inter-subtype variation. A/H3N2 demonstrated the most dramatic threshold evolution: pre-pandemic curves showed a bimodal promotion pattern at low (~6.46 km; 95% CI: 6.14–16.17 km) and high (~14.98 km; 95% CI: 6.34–16.16 km) visibility ranges (Figure 4A), with inhibition at intermediate levels. During the pandemic, this relationship simplified to promotion occurring only above 15.79 km (95% CI: 15.56–16.07 km). The post-pandemic period exhibited a complete inversion, with transmission promotion confined to a narrow 15–17 km range and inhibition or neutrality elsewhere. A/H1N1 visibility responses showed progressive simplification across pandemic phases (Figure 4D). Pre-pandemic promotion occurred within discrete ranges (6.72–9.60 km and >15.01 km; 95% CI: 6.51–15.21 km), while pandemic-period effects shifted to inhibition below 9.93 km (95% CI: 9.87–19.23 km) with only marginal promotion above 15.41 km. Post-pandemic effects consolidated into peak promotion within the 15–17 km visibility band. B/Victoria exhibited the most stable visibility-response profile, with pre-pandemic inhibition at 6.78–10.72 km and weak promotion at 10.74–16.30 km. During the pandemic, strong promotional effects emerged within the 11.85–16.36 km range (Figure 4F). However, the post-pandemic period showed a notable reversal, with visibility >15.41 km becoming inhibitory while lower visibility values became promotional, suggesting fundamental shifts in the environmental sensitivity of this lineage.

## 4. Discussion

This study investigated the temporal evolution of influenza virus subtype-specific positivity rates in Southern China across pre-, during-, and post-COVID-19 pandemic periods and their complex relationships with environmental drivers. We integrated XG-Boost modeling with SHAP analysis to quantify the relative contributions of environmental factors to transmission dynamics, and employed GAMs to characterize the nonlinear relationships and critical threshold points governing subtype-specific risk transitions. Our findings reveal pronounced heterogeneity in environmental sensitivities among influenza subtypes across pandemic phases, demonstrating that individual A subtypes (H3N2 and H1N1) and B lineage (Victoria) exhibit distinct and temporally variable responses to environmental conditions. While previous investigations have primarily examined aggregate influenza-environment associations without subtype differentiation [18], our approach advances this field by elucidating the differential environmental dependencies of specific viral lineages and their dynamic evolution during periods of major epidemiological disruption.

Our analysis reveals that the COVID-19 pandemic fundamentally disrupted established influenza transmission patterns through complex interactions between environmental drivers and behavioral modifications, with each subtype exhibiting distinct adaptive responses across pandemic phases. Environmental driver redistribution during pandemic disruption demonstrated subtype-specific vulnerabilities to altered transmission contexts. A/H3N2 experienced the most pronounced environmental dependency shifts, with PM_10_ contributions surging from 9.18% to 30.37% during lockdown periods—reflecting enhanced indoor aerosol transmission under confinement conditions—while visibility contributions plummeted from 37.26% to 7.42% [19, 20]. This redistribution coincided with absolute humidity threshold shifts from 17.08 g/m³ pre-pandemic to 14.93 g/m³ during pandemic restrictions, suggesting that modified indoor environmental conditions fundamentally altered optimal transmission parameters [21–23]. The dramatic visibility contribution recovery to 58.05% post-pandemic, exceeding prepandemic levels, indicates that accumulated susceptibility effects amplified environmental transmission drivers following restriction relaxation.

A/H1N1 demonstrated the most severe pandemic-period suppression, with temperature contributions declining by 17.66% points while humidity and PM_10_ dependencies increased substantially. This environmental driver redistribution, coupled with near-zero detection rates, suggests that pandemic-period modifications disproportionately disrupted this subtype's temperature-dependent transmission mechanisms [24]. The subsequent dramatic summer positivity recovery to 4.325%—nearly five-fold higher than pre-pandemic levels—with visibility promotion consolidating into the 15–17 km band, indicates refined environmental adaptation following pandemic-period selection pressures [25–28]. B/Victoria exhibited remarkable environmental stability throughout pandemic phases, maintaining consistent sea-level pressure dependencies (25.52% pre-pandemic, 24.34% during pandemic, and 36.36% post-pandemic) while showing progressive declines in humidity and PM_10_ contributions. This stability, combined with consistently low-amplitude seasonal variation, suggests that pressure-driven transmission mechanisms conferred resilience against pandemic-period environmental disruptions [29]. The post-pandemic consolidation toward atmospheric pressure dependance (36.36% contribution) indicates evolutionary refinement toward this lineage's core transmission niche.

Threshold dynamics reveal adaptive plasticity in environmental sensitivity patterns. A/H3N2's absolute humidity thresholds demonstrated systematic drift across pandemic phases (17.08 g/m³ → 14.93 g/m³ → 19.60 g/m³), reflecting dynamic adaptation to changing environmental contexts. Similarly, visibility thresholds for all subtypes showed complex temporal evolution, with A/H1N1 transitioning from bimodal promotion patterns to consolidated 15–17 km band optimization, suggesting selection for specific atmospheric clarity conditions that balance UV resistance with transmission efficiency. These findings demonstrate that influenza subtypes possess distinct environmental adaptation capacities that become apparent under extreme epidemiological disruption. The differential recovery patterns—A/H3N2's overshoot response, A/H1N1's dramatic rebound, and B/Victoria's stable consolidation—suggest that pandemic-period environmental pressures may have lasting effects on subtype-specific transmission ecology, with implications for future seasonal prediction models and public health preparedness strategies.

This study has several limitations. First, it relies on observational surveillance and environmental data and cannot fully account for potential confounders, such as vaccination coverage, public health interventions, and human behavior; thus, associative findings do not imply causality [30]. As Shaman and Kohn [31] have noted, explaining influenza seasonality via environmental drivers remains a hypothesis requiring experimental validation. Second, although our models included key environmental variables, they may have omitted important biological factors (e.g., viral genetic variation and population-level immunity dynamics), and SHAP values represent average effects susceptible to data distribution and multicollinearity influences. Third, our study setting in a subtropical region may limit generalizability to other climates [32]. Fourth, this analysis did not incorporate COVID‑19–era public health interventions (e.g., mask mandates, mobility restrictions, and lockdown policies), which have demonstrated substantial impacts on respiratory virus transmission; omitting these measures may bias the estimated effects of climatic drivers, particularly during 2020–2022. Fifth, the post-pandemic period analyzed (November 2022–May 2024) encompasses only ~ 18 months (≈78 weekly observations), which, given rapid viral evolution and shifting population immunity, may yield unstable estimates and wider confidence intervals for climate‑influenza associations. Future work should integrate additional data sources (including viral genomics and intervention indices) and employ experimental or natural experimental designs to validate the environmental mechanisms observed.

In summary, this study elucidates the dynamic relationships between environmental drivers and influenza A subtypes and B lineage in Southern China across successive COVID‑19 phases. Our findings indicate that strict NPIs nearly eliminated certain subtypes, while relaxation led to rebounds driven by immunity gaps and altered contact patterns. Concurrently, the effects of environmental factors—PM_10_, visibility, temperature, absolute humidity, and sea‑level pressure—on viral transmission exhibited pronounced phase‑specific and nonlinear behaviors. These insights advance our understanding of influenza transmission mechanisms and provide empirical support for more targeted early‑warning systems and public‑health interventions.

## Data Availability

Weekly influenza surveillance data (2011–2024) from the China National Influenza Center are available upon reasonable request. Meteorological and air-pollution data from Visual Crossing Weather and the National Tibetan Plateau Data Center are publicly accessible via their respective portals. All analysis scripts and derived datasets are available from the corresponding author upon reasonable request.

## Supporting Information

Supporting Information 1 A/H1N1 positive rate and corresponding SHAP values and raw data for climate factors.

Supporting Information 2 A/H3N2 positive rate and corresponding SHAP values and raw data for climate factors.

Supporting Information 3 B/Victoria positive rate and corresponding SHAP values and raw data for climate factors.

Supporting Information 4 Figure S1: Subtype-specific feature importance (mean |SHAP|%) of environmental drivers for influenza A subtypes and B lineage. Table S1: Evaluation metrics for XGBoost model training. Table S2: GAM smoothing parameter settings for each era of COVID-19 by influenza A subtypes and B lineage. Table S3: Differential analysis of positive rates of Influenza A subtypes and B lineage across distinct periods. Table S4: Differential analysis of environmental factors across pandemic phases. Table S5: Contribution (%) of environmental factors across different periods for subtypes (lineage). Table S6: Statistical description of the analyzed data.

Supporting Information 5 The average positive rate of each subtype in different periods of the epidemic season.

Supporting Information 6 Thresholds for risk conversion of subtype infections induced by key environmental factors.
