# Supplementary material for: Temporal Shifts in Climate-Influenza Dynamics: A Multi-Subtype Analysis in Southern China Spanning the COVID-19 Era
Source: Transbound Emerg Dis. 2025 Aug 11;2025:5581162. doi: 10.1155/tbed/5581162 (PMC12360877; doi:10.1155/tbed/5581162)
Supplement: Supporting Information 4 — Figure S1: Subtype-specific feature importance (mean |SHAP|%) of environmental drivers for influenza A subtypes and B lineage. Table S1: Evaluation metrics for XGBoost model training. Table S2: GAM smoothing parameter settings for each era of COVID-19 by influenza A subtypes and B lineage. Table S3: Differential analysis of positive rates of Influenza A subtypes and B lineage across distinct periods. Table S4: Differential analysis of environmental factors across pandemic phases. Table S5: Contribution (%) of environmental factors across different periods for subtypes (lineage). Table S6: Statistical description of the analyzed data. [file 5581162.f4.docx]

**Supplementary information**

# Temporal Shifts in Climate-Influenza Dynamics: A Multi-Subtype Analysis in Southern China Spanning the COVID-19 Era

Lirong Zhang^1^, Jiajia Lin^1^, Jiaxiong Zheng^1^, Yixiao Niu^1^, Fancun Meng^1^, Suyang Liu^1^, Qiaocheng Chang^1^, ZichengCao^1,2,3,4^

**Corresponding to:**

Dr. Zicheng Cao, School of Public Health, Shantou University, Shantou, P.R. China.

Email: zichengcao@stu.edu.cn

**Affiliation:**

. School of Public Health, Shantou University, Shantou 515041, P.R. China

2. State Key Laboratory of Public Big Data, Guizhou University, Guiyang 550025, P.R. China

3. School of Public Health (Shenzhen), Shenzhen Campus of Sun Yat-sen University, Shenzhen 518107, P.R. China

4. Shenzhen Key Laboratory of Pathogenic Microbes and Biosafety, Shenzhen Campus of Sun Yat-sen University, Shenzhen 518107, P.R. China

## Materials & Methods

The *AH* (Absolute Humidity) calculation formula used in the data is based on the Clausius-Clapeyron equation, which is expressed as follows:

$AH=\frac{6.112*e^{\frac{(17.67*T)}{(T+243.5)}}*RH*2.1674)}{(273.15+T)}$

where $T$ represents the air temperature in degrees Celsius (°C), and $RH$ is the relative humidity as a percentage. The exponential term involves temperature and is derived from the Clausius-Clapeyron equation, which models the saturation vapor pressure. The constants in the formula originate from the Magnus-Tetens approximation and other empirical relationships that describe the behavior of water vapor in the air.

## Results

Table S1 Evaluation Metrics for XG-Boost Model Training

| Subtypes (Lineage) | *R²* | *MSE* | *RMSE* | *MAE* |
| --- | --- | --- | --- | --- |
| B/Victoria | 0.9999 | 0.0030 | 0.0544 | 0.0365 |
| A/H3N2 | 0.9997 | 0.0166 | 0.1288 | 0.0916 |
| A/H1N1 | 0.9999 | 0.0031 | 0.0554 | 0.0403 |

Table S2 GAM Smoothing Parameter Settings for Each Era of COVID-19 by

Influenza A Subtypes and B Lineage

| Subtypes(Lineage) | Period | No. of Splines | Lambda (λ) |
| --- | --- | --- | --- |
| B/Victoria | Pre-COVID | 90 | 0.2 |
|  | COVID-19 | 80 | 0.6 |
|  | Post-COVID | 80 | 0.6 |
| A/H3N2 | Pre-COVID | 150 | 0.1 |
|  | COVID-19 | 14 | 0.6 |
|  | Post-COVID9 | 90 | 0.5 |
| A/H1N1 | Pre-COVID | 23 | 0.7 |
|  | COVID-19 | 80 | 0.6 |
|  | Post-COVID | 99 | 0.1 |

*Noted: No. of splines, number of basic functions employed for smoothing; λ (lambda), smoothing parameter governing the degree of curve flexibility in fitted relationships.*

Table S3 Differential Analysis of Positive Rates of Influenza A Subtypes and B Lineage Across Distinct Periods

| Epidemic Season of Subtypes(Lineage) | Period | Statistic | *P*-Value |
| --- | --- | --- | --- |
| A/H3N2 (summer) | Pre- vs COVID | 11723.00 | ＜0.00 |
|  | Pre- vs Post- | 3322.00 | 0.87 |
|  | COVID vs Post- | 652.00 | 0.0010 |
| A/H3N2 (winter) | Pre- vs COVID | 15277.50 | ＜0.00 |
|  | Pre- vs Post- | 3853.00 | ＜0.00 |
|  | COVID vs Post- | 366.00 | ＜0.00 |
| A/H1N1(summer) | Pre- vs COVID | 14745.00 | ＜0.00 |
|  | Pre- vs Post- | 7111.00 | 0.064 |
|  | COVID vs Post- | 815.00 | ＜0.00 |
| B/Victoria (summer) | Pre- vs COVID | 3562.00 | ＜0.00 |
|  | Pre- vs Post- | 4105.00 | 0.0002 |
|  | COVID vs Pos-t | 2242.00 | 0.03 |

*Noted: Three-group comparisons performed using Kruskal-Wallis test or ANOVA; pairwise comparisons conducted via Mann-Whitney U test, with statistical significance set at P<0.05.*

Table S4 Differential Analysis of Environmental Factors Across Pandemic Phases

| Factor | Pre- vs COVID  *P*-Value | Pre- vs Post-  *P*-Value | COVID vs Post-  *P*-Value |
| --- | --- | --- | --- |
| Temperature | 0.190 | 0.132 | 0.032 |
| Feels like | 0.183 | 0.154 | 0.033 |
| Solar radiation | 0.137 | 0.117 | 0.737 |
| Solar energy | 0.136 | 0.118 | 0.741 |
| UV-index | 0.220 | 0.717 | 0.296 |
| DTR | 0.022 | 0.536 | 0.271 |
| Humidity | 0.370 | 0.000 | 0.011 |
| windspeed | 0.010 | 0.823 | 0.124 |
| Wind direction | 0.002 | 0.038 | 0.411 |
| Cloud cover | 0.001 | 0.160 | 0.292 |
| Visibility | 0.000 | 0.000 | 0.000 |
| Absolute humidity | 0.289 | 0.047 | 0.016 |
| Sea level pressure | 0.000 | 0.000 | 0.102 |
| PM_2.5_ | 0.000 | 0.000 | 0.055 |
| PM_10_ | 0.000 | 0.000 | 0.069 |
| NO_2_ | 0.000 | 0.000 | 0.259 |

*Noted: Comparisons between three groups performed using Kruskal-Wallis test or ANOVA; pairwise comparisons conducted via Mann-Whitney U test, with statistical significance defined at P<0.05.*


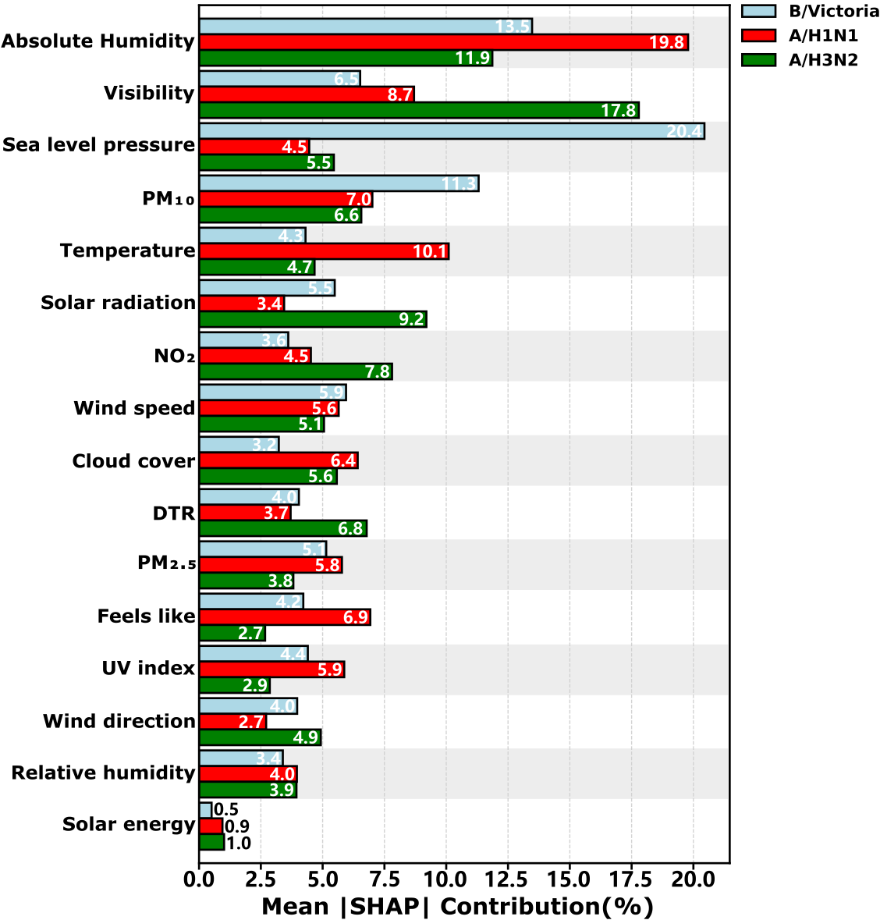


Figure S1 Subtype-specific feature importance (mean |SHAP| %) of environmental drivers for Influenza A subtypes and B lineage

Table S5 Contribution (%) of Environmental Factors across Different Periods for Subtypes (Lineage)

| Environmental factors | Pre-COVID | | | COVID-19 | | | Post-COVID | | |
| --- | --- | --- | --- | --- | --- | --- | --- | --- | --- |
|  | A/H1N1 | A/H3N2 | B/Victoria | A/H1N1 | A/H3N2 | B/Victoria | A/H1N1 | A/H3N2 | B/Victoria |
| Temperature | 28.02 | 1.32 | 4.37 | 10.36 | 6.94 | 3.08 | 16.67 | 1.90 | 5.53 |
| Feels like | 14.44 | 1.99 | 3.30 | 12.97 | 3.32 | 2.63 | 7.85 | 0.49 | 2.31 |
| Humidity | 2.21 | 0.66 | 8.37 | 2.30 | 1.58 | 5.85 | 8.43 | 2.69 | 5.32 |
| Wind direction | 0.93 | 1.07 | 2.11 | 0.65 | 0.68 | 5.44 | 3.14 | 1.38 | 1.17 |
| Wind speed | 1.09 | 0.47 | 2.44 | 6.33 | 6.81 | 7.25 | 6.92 | 0.75 | 4.08 |
| Sea level pressure | 2.38 | 5.18 | 25.52 | 0.84 | 4.09 | 24.34 | 6.42 | 6.27 | 36.36 |
| Cloud cover | 3.68 | 3.03 | 0.78 | 2.15 | 3.35 | 1.17 | 5.70 | 0.23 | 0.22 |
| Visibility | 4.65 | 37.26 | 2.63 | 15.02 | 7.42 | 6.42 | 7.02 | 58.05 | 7.72 |
| Solar radiation | 5.83 | 9.00 | 2.86 | 1.23 | 4.44 | 1.70 | 2.24 | 1.51 | 7.50 |
| Solar energy | 0.11 | 2.17 | 2.23 | 0.61 | 2.89 | 0.80 | 0.52 | 0.78 | 0.80 |
| UV-index | 6.38 | 4.73 | 0.71 | 1.01 | 6.25 | 0.21 | 8.46 | 2.82 | 3.09 |
| DTR | 1.31 | 0.53 | 0.32 | 3.17 | 1.50 | 0.56 | 0.36 | 1.20 | 0.56 |
| Absolute humidity | 13.84 | 1.13 | 13.88 | 18.59 | 8.66 | 6.79 | 3.78 | 5.00 | 0.49 |
| PM_2.5_ | 6.87 | 2.99 | 1.06 | 6.11 | 6.90 | 10.66 | 6.74 | 3.20 | 11.52 |
| PM_10_ | 3.18 | 9.18 | 25.14 | 17.89 | 30.37 | 21.72 | 9.44 | 3.95 | 11.35 |
| NO^2^ | 5.07 | 19.28 | 4.27 | 0.78 | 4.81 | 1.39 | 6.30 | 9.77 | 1.98 |

Table S6 Statistical Description of the Analyzed Data

| Factors | Min. | 25th percentile | Median | 75th percentile | Max. | Mean |
| --- | --- | --- | --- | --- | --- | --- |
| A/H3N2 positive rate (%) | 0.00 | 2.00 | 2.00 | 2.00 | 47.00 | 5.00 |
| A/H1N1 positive rate (%) | 0.00 | 0.00 | 0.00 | 0.00 | 45.00 | 3.00 |
| B/Victoria positive rate (%) | 0.00 | 0.00 | 0.00 | 0.00 | 28.00 | 3.00 |
| Temperature (℃) | 2.84 | 19.21 | 19.21 | 19.21 | 30.21 | 18.41 |
| Feels like (℃) | 0.47 | 19.3 | 19.3 | 19.3 | 35.43 | 18.91 |
| Relative humidity (%) | 51.11 | 76.19 | 76.19 | 76.19 | 86.54 | 75.2 |
| Wind speed (kph) | 12.26 | 17.01 | 17.01 | 17.01 | 23.27 | 17.17 |
| Wind direction (degrees) | 83.29 | 141.29 | 141.29 | 141.29 | 214.1 | 141.78 |
| Sea level pressure (mb) | 866.02 | 1008.74 | 1008.74 | 1008.74 | 1032.76 | 973.68 |
| Cloud cover (%) | 12.01 | 61.47 | 61.47 | 61.47 | 87.12 | 59.59 |
| Absolute humidity (%) | 3.88 | 12.13 | 12.13 | 12.13 | 22.35 | 12.88 |
| Visibility (km) | 6.04 | 11.33 | 11.33 | 11.33 | 19.43 | 11.88 |
| Solar radiation (W/m2) | 62.21 | 175.42 | 175.42 | 175.42 | 289.59 | 171.39 |
| Solar energy (MJ/m2) | 5.37 | 15.14 | 15.14 | 15.14 | 25.01 | 14.8 |
| UV index | 2.63 | 6.35 | 6.35 | 6.35 | 9.31 | 6.21 |
| DTR (℃) | 4.76 | 8.69 | 8.69 | 8.69 | 14.37 | 8.78 |
| PM2.5(μg/m³) | 12.25 | 36.06 | 36.06 | 36.06 | 103.38 | 38.86 |
| PM10(μg/m³) | 22.62 | 59.1 | 59.1 | 59.1 | 144.87 | 62.6 |
| NO2(μg/m³) | 11.15 | 24.79 | 24.79 | 24.79 | 58.98 | 25.63 |
